# Aporphine Alkaloids from the Leaves of *Phoebe grandis* (Nees) Mer. (Lauraceae) and Their Cytotoxic and Antibacterial Activities

**DOI:** 10.3390/molecules18088994

**Published:** 2013-07-29

**Authors:** Hanita Omar, Najihah Mohd. Hashim, Asdren Zajmi, Noraziah Nordin, Siddiq Ibrahim Abdelwahab, Ainnul Hamidah Syahadah Azizan, A. Hamid A. Hadi, Hapipah Mohd Ali

**Affiliations:** 1 Chemistry Department, Faculty of Science, University of Malaya, 50603 Kuala Lumpur, Malaysia; E-Mails: ainnul_azizan@yahoo.com (A.H.S.A.); ahamid@um.edu.my (A.H.A.H.); 2 Centre for Foundation Studies in Sciences, University of Malaya, 50603 Kuala Lumpur, Malaysia; 3 Department of Pharmacy, Faculty of Medicine, University of Malaya, 50603 Kuala Lumpur, Malaysia; E-Mails: najihahmh@gmail.com (N.M.H.); asza22@yahoo.com (A.Z.); aziereality@yahoo.com (N.N.); 4 Medical Research Center, Faculty of Medicine, Jazan University, Jazan 45041, Saudi Arabia; E-Mail: siddigroa@yahoo.com

**Keywords:** *Phoebe grandis*, Lauraceae, aporphine alkaloid, cytotoxic, antibacterial

## Abstract

The oxoaporphine alkaloid lysicamine (**1**), and three proaporphine alkaloids, litsericinone (**2**), 8,9,11,12-tetrahydromecambrine (**3**) and hexahydromecambrine A (**4**) were isolated from the leaves of *Phoebe grandis* (Nees) Merr. (Lauraceae). Compounds **2** and **3** were first time isolated as new naturally occurring compounds from plants. The NMR data for the compounds **2**–**4** have never been reported so far. Compounds **1** and **2** showed significant cytotoxic activity against a MCF7 (human estrogen receptor (ER+) positive breast cancer) cell line with IC_50_ values of 26 and 60 µg/mL, respectively. Furthermore, *in vitro* cytotoxic activity against HepG2 (human liver cancer) cell line was evaluated for compounds **1**–**4** with IC_50_ values of 27, 14, 81 and 20 µg/mL, respectively. Lysicamine (**1**) displayed strong antibacterial activity against *Bacillus subtilis* (B145), *Staphylococcus aureus* (S1434) and *Staphylococus epidermidis* (a clinically isolated strain) with inhibition zones of 15.50 ± 0.57, 13.33 ± 0.57 and 12.00 ± 0.00 mm, respectively. However, none of the tested pathogenic bacteria were susceptible towards compounds **2** and **3**.

## 1. Introduction

Cancer is the most common and fatal disease and accounted for 7.6 million deaths (about 13% of all deaths) in 2008. Deaths from cancer worldwide are projected to continue to rise to over 11 million in 2030 [1]. Breast cancer is one of the main life-treatening diseases that a woman may have to face during her lifetime and is the most widespread [2].

About 60% of anticancer drugs used nowadays are obtained from natural sources [3]. Out of total 250,000 plant species existing on Earth approximately one thousand have known anticancer potential. A large number of plant species have been screened through bioassays in the search for novel plant-based anticancer drugs [4]. The present investigation aimed to assess the cytotoxic potential of alkaloids isolated from leaves of *Phoebe grandis* (Nees) Merr. (Lauraceae) on selected cell lines.

Over the last decade, antibiotics which were considered to be miracle drugs have been losing their effectiveness as pathogens evolve resistance against them [5]. Antibacterial resistance continues to grow quickly among key pathogens such as *Staphylococcus epidermidis* (a Gram-positive bacterium)—which is a part of human skin flora and also can be found in the mucous membranes and in animals [6]; *Staphylococcus aureus* (a Gram-positive bacterium) is frequently found in the human respiratory tract and on the skin, and it is estimated that 20% of the human population are long-term carriers of *S. aureus* [7,8]; *Bacillus subtilis* (a Gram-positive bacterium) is known as the ‘hay bacillus or grass bacillus’, commonly found in soil, and more evidence suggests that *B. subtilis* is a normal gut commensal in humans [9]; *Pasteurella multocida*, a Gram-negative bacterium, causes a range of diseases in mammals and birds, including fowl cholera in poultry [10,11] and *Enterobacter cloacae*, a Gram-negative bacterium, is commonly found in infections among burn victims, immunocompromised patients, and patients with malignancies [12]. Development of new antibacterial agents is imperative. The increased prevalence of antibiotic-resistant bacteria due to the extensive use of antibiotics may render the current antimicrobial agents insufficient to control some bacterial diseases. Therefore, the search for new promising drugs and especially plant- drug derived may give a solution for the drug resistance crisis.

Several medicinal plants have been extensively studied in order to find more effective and less toxic compounds. Lauraceae is one of the major families of plants, which is widespread in the tropical and subtropical regions, particularly in tropical America, Africa and Southeast Asia [13]. The Lauraceae or laurel family is known as “*Medang*” or “*Tejur*” by the Malays, and comprises a group of flowering plants included in the order Laurales; it consists of 55 genera and over 2,500 (perhaps as many as 4,000) species worldwide and about 16 genera and 213 species are found in Malaysia [14,15]. In Malaysia the species are largely montane. *Phoebe* species commonly grow most abundantly in Borneo and Peninsular Malaysia from central Perak to Malacca. *Phoebe* species are rich in alkaloidal constituents [16,17,18] and have been reported to contain the rare propaorphine-tryptamine dimers [19,20], aporphines [19,21,22,23,24,25], proaporphine [19,25,26,27] and oxoaporphines [21,25,27] compounds. An interesting aporphine alkaloid named laurolitsine isolated from the stems of *P. formosana* (Hayata) has been used as a starting material to prepare bioactive phenanthrene alkaloids [28]. Some species of *Phoebe* also were reported in China, Indonesia, Indochina, Japan, The Philippines and the Malay Peninsula [15] to be useful to treat several diseases thanks to their antidiabetic, antibacterial and antifungal activities [24]. Aqueous extract of berries of *P. lanceolata* is an important remedy and has been used in the traditional medicine system in India for wounds and sores, whereas ethanolic extracts of *P. lanceolata* stem bark were evaluated for their antibacterial activity against five bacterial species: *Staphylococcus aureus* (along with ten hospital-derived strains), *Staphylococcus mutans*, *Staphylococcus epidermidis*, *Escherichia coli* and *Klebsiella pneumoniae* with MIC range of 50–100 µg/mL [29].

**Figure 1:**
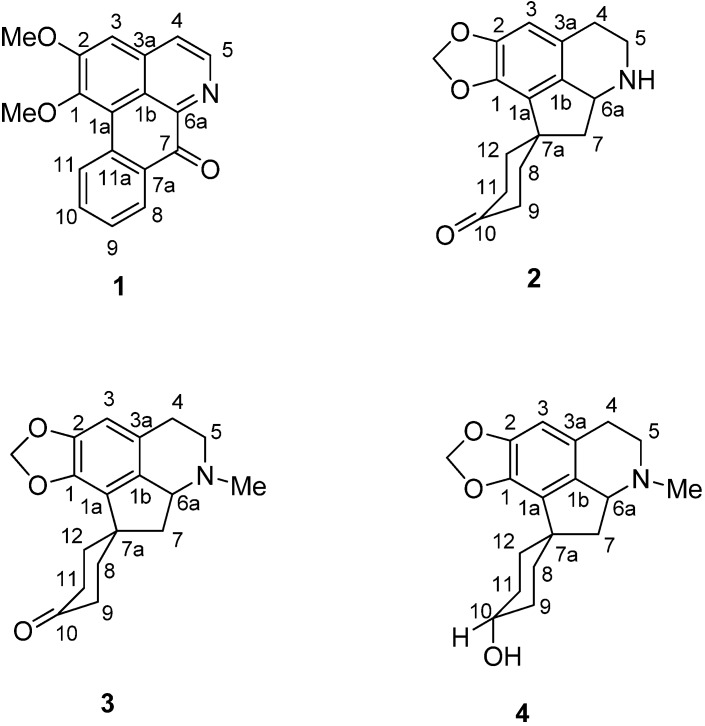
Alkaloids **1**–**4** isolated from *Phoebe grandis* (Nees) Merr. (Lauraceae).

*Phoebe grandis* is locally known in Malaysia as “*medang ketanah or tanah*” [30,31]. A proaporphine alkaloid which occurs in *P. grandis* is used as a precursor in the synthesis of aporphines and proaporphine-tryptamines alkaloids [25]. These alkaloids are known for their unique pharmacological activities, as demonstrated by liriodenine which shows antitumor, antibacterial, and antifungal activities [27]. Biological screening on the crude alkaloidal extract of the leaves of *Phoebe grandis* for antiplasmodial activity has shown positive results too: IC_50_ < 8 µg·mL^−1^ [32]. Although, most of the species of *Phoebe* are rich in alkaloidal contents, a few species are also reported as alkaloid-free [33]. We have launched a chemical investigation on the extract. Further investigation of the leaves has now led to the isolation of a known oxoaporphine alkaloid, lysicamine (**1**), and three proaporphine alkaloids, litsericinone (**2**), 8,9,11,12-tetrahydromecambrine (**3**) and hexahydro-mecambrine A (**4**) (Figure 1). Compounds **2** and **3** were isolated for the first time as natural compounds. Compound **4** has also been isolated from *Phoebe scortechinii* [34] and was previously synthesized by Nakasato [35].

The objective of this research was to isolate and identify bioactive compounds from *Phoebe grandis* (Nees) Merr. (Lauraceae) with potential cytotoxicity against the MCF7 and HepG2 cell lines and test their antibacterial activities.

## 2. Results and Discussion

### 2.1. Compound characterization

Lysicamine (**1**) was obtained as a yellow amorphous solid. 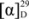 = −25 (c = 0.00008, CHCl_3_). The mass spectrum showed the [M+H]^+^ peak at *m/z* = 292.0963, which corresponds to a molecular formula of C_18_H_13_NO_3_. (calcd. for C_18_H_14_NO_3_, 292.0929). Other significant fragmentations observed were at *m/z* 277, which may be attributed to the loss of a CH_3_ molecule, [M−15]^+^. The UV spectrum showed absorption maxima at 236, 267, 360 and 396 nm, indicating the existence of a highly unsaturated oxoaporphine chromophore [36,37]. The IR spectrum showed a conjugated ketone peak at 1665 cm^−1^ [37,38]. The ^1^H-NMR spectrum showed two distinct methoxyl peaks at δ 4.00 and δ 4.08, which were probably situated at C-1 and C-2. H-3 appeared as a singlet at δ 7.21. Two doublets (*J* = 5.2 Hz) typical of the H-4 and H-5 signals of an oxoaporphine were observed at δ 7.78 and δ 8.88, respectively. The H-5 proton was deshielded by the neighbouring N atom. A very downfield signal at δ 9.16 appeared as doublet of doublet with *J_1_* = 8.4 and *J_2_* = 0.7 belongs to H-11. In addition, a doublet of doublet peak was observed at δ 8.57 (1H, *dd*, *J_1_* = 7.9 Hz, *J_2_* = 1.4 Hz; H-8) which experienced a deshielding effect from the neighbouring C-7 carbonyl group. The peak appeared as doublet-triplet at δ 7.75 with *J_1_ =* 8.52 Hz and *J_2_* = 1.64Hz was assigned for H-9 whereas H-10 resonated at δ 7.56 as doublet-triplet with *J_1_ =* 8.52 Hz and *J_2_* = 1.64 Hz. The ^13^C-NMR spectrum gave a total of eighteen carbons which validated the molecular formula of C_18_H_13_NO_3_. Analysis of the ^13^C-NMR spectrum gave nine quaternary carbons. Hence, compound **1** is an oxoaporphine alkaloid and in fact it was identified as lysicamine by the full agreement of the ^1^H- and ^13^C-NMR data of **1** with the literature values for that compound [39,40].

Litsericinone (**2**) was isolated as a yellow amorphous solid, 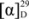 = −25 (c = 0.00004, CHCl_3_). This proaporphine alkaloid exhibited an [M+H]^+^ peak in the LCMS-IT-TOF ESI (positive mode) mass spectrum at *m/z* 286.1421 which correlated to the molecular formula of C_17_H_19_NO_3_ (calcd. for C_17_H_20_NO_3_, 286.1432). The UV spectrum revealed three peaks at 300, 236 and 207 nm, which indicated the existence of a conjugated system [37]. The IR spectrum revealed a very significant carbonyl absorption at 1712.52 cm^−1^. In addition, the presence of a methylenedioxyl group was proven by its characteristic absorption peaks at 1248.37 cm^−1^ and 934.90 cm^−1^, which indicate asymmetric C-O-C stretching.

The ^1^H-NMR spectrum showed one aromatic proton singlet at δ 6.51 which may be ascribed to H-3. H-6a resonated at δ 4.26 (*dd*, *J*, *J’* = 9.8, 6.9Hz). The methylenedioxyl protons appeared as a pair of doublets at δ 5.89 and δ 5.86 with *J* = 1.3 Hz. The aliphatic protons of ring D resonated as multiplets between δ 1.91 to 2.68, while H-7 appeared as multiplet at δ 2.73 and δ 1.87 as showed in HSQC spectrum. The above observations were reinforced by COSY experiment which showed correlations between vicinal protons; H4_ax_/H5_ax_, H4_eq_/H5_eq_, H5_ax_/H4_ax_, H5_eq_/H4_eq_, H6a/H7_eq_, H8_eq_/H9_eq_, H9_eq_/H8_eq_, H11_ax_/H12_ax_ and H12_ax_/H11_ax_. Interestingly, H6a revealed correlation to H7_eq_ suggesting the possibility that H6a is in an equatorial configuration.

**Figure 2:**
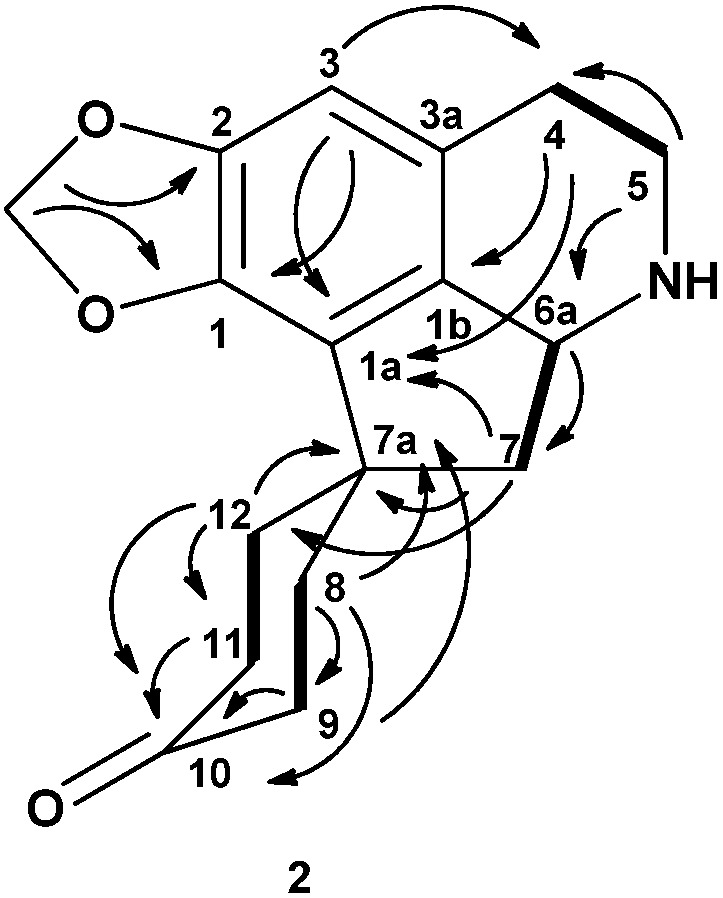
Key HMBC (→) and COSY (▬) correlations of litsericinone (**2**).

The ^13^C-NMR spectrum of litsericinone (**2**) showed the presence of seventeen carbon atoms, whereas the DEPT experiment displayed the presence of seven methylenes, a methylenedioxyl and two methine signals. The ^13^C-NMR spectrum revealed the C-10 carbonyl peak at δ 211.12 and the quarternary carbon showed five signal peaks between δ 123.91 to 148.90. The characteristic proaporphine quarternary C-7a spirocarbon appeared at δ 46.14. The complete assignments of all protons and carbons (Table 1) were aided by the HSQC and HMBC experiments. The proximity of H6a and H7_eq_ was confirmed by the HMBC correlation peaks between H5_eq_/C6a and H7_eq_/C6a. Figure 2 shows the HMBC and COSY correlation of litsericinone (**2**).

**Table 1:** ^13^C-NMR (150 MHz), ^1^H-NMR (600 MHz) and HMBC spectral data of litsericinone, (**2**) in CDCl_3_ (*δ* in ppm, *J* in Hz).

Position	^13^C (δ, CDCl_3_)	Type	^1^H (*J*, Hz)	HMBC (^2^*J*, ^3^*J*)
1	148.9	C_q_	-	
1a	141.1	C_q_	-	
1b	123.9	C_q_	-	
2	141.1	C_q_	-	
3	106.9	CH	6.51 s	C1, C1a, C2, C4
3a	126.7	C_q_	-	
4	25.1	CH_2_	2.87 m (ax)	C3, C1b, C1a
			2.75 m (eq)	C3, C1b, C1a
5	43.9	CH_2_	3.55 m (ax)	C4, C6a, C1b
			3.16 m (eq)	C4, C6a
6a	56.8	CH	4.26 dd	C5, C7
			(*J* = 6.9 Hz,	
			*J =* 9.8 Hz)	
7	44.1	CH_2_	2.73 m (ax)	C3, C3a, C1a
			1.87 m (eq)	C12, C7a, C6a
7a	46.2	C_q_	-	-
8	38.5	CH_2_	2.68 m (ax)	C10
			2.41 m (eq)	C9, C7a, C10
9	36.2	CH_2_	2.50 m (ax)	C8, C7a, C10
			1.91 m (eq)	C11, C7a, C10
10	211.1	C=O	-	-
11	38.9	CH_2_	2.46 m (ax)	C10
			2.45 m (eq)	C12, C7a, C10
12	34.3	CH_2_	2.10 m (ax)	C9, C7, C7a, C3a, C10
			2.00 m (eq)	C9, C7a, C3a, C10
Methylenedioxy (O-CH_2_-O)	100.9	CH_2_	5.89 d (*J* = 1.3 Hz)	C1,C2
			5.86 d (*J* = 1.3 Hz)	C1,C2

8,9,11,12-Tetrahydromecambrine, (**3**) was isolated as a yellow amorphous solid, 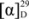 = −25 (c = 0.00004, CHCl_3_). The LCMS-IT-TOF mass spectrum of this proaporphine type of alkaloid showed a [M+H]^+^ peak at *m/z* = 300.1596 which correlated to the molecular formula C_18_H_21_NO_3_ (calcd. for C_18_H_22_NO_3_, 300.1521). The UV spectrum showed an absorption peak at 203 nm. The IR spectrum showed a very significant carbonyl absorption peak at 1712.80 cm^−1^ due to C=O stretching vibrations. The presence of the methylenedioxyl group was proven by its characteristic absorption peaks at 1254.45 and 944.84 cm^−1^, which indicate the asymmetric C-O-C stretching.

**Figure 3:**
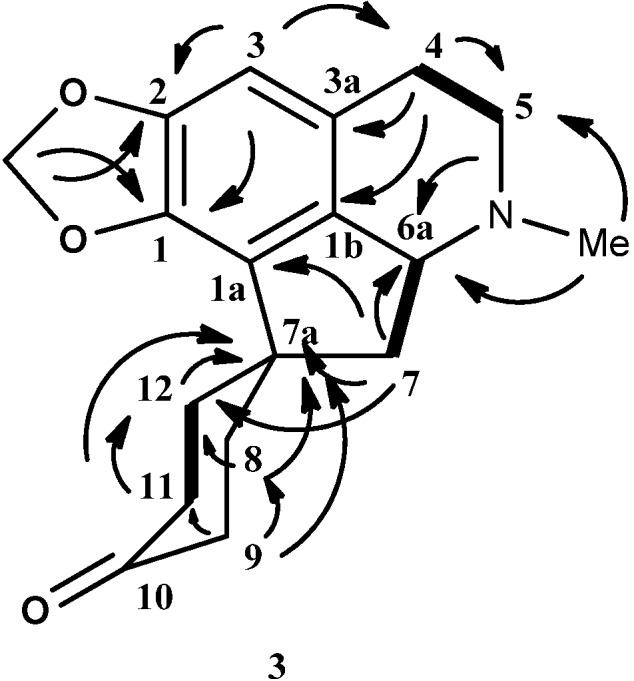
Key HMBC (→) and COSY (▬) of 8,9,11,12-tetrahydromecambrine (**3**).

The ^1^H-NMR spectrum displayed a pair of doublet peaks at δ 5.88 (d, *J* = 1.2 Hz) and 5.83 (d, *J* = 1.2 Hz) that correspond to the methylenedioxyl group at positions C-1 and C-2. A singlet peak appeared at δ 6.49 representing a proton attached to a benzene ring at the C-3 position. There is a strong singlet peak at δ 2.39 indicating a N-methyl group. The aliphatic protons appeared between δ 1.70 to 3.30. These peaks supported were by the COSY experiment that showed correlation peaks between H5_ax_/H4_ax_, H4_ax_/H5_ax_, H6a/H7_ax_, H7_ax_/H6a, H6a/H7_eq_, H7_eq_/H6a, H11/H12_ax_, H12_ax_/H11, H11/H12_eq_ and H12_eq_/H11. The ^13^C-NMR spectrum of **3** showed the presence of eighteen carbons and the DEPT experiment showed the presence of seven methylene carbons (CH_2_) and one methylenedioxyl group. The carbonyl group at C-10 position resonated at δ 211.65 and the N-methyl group at δ 43.47. The quaternary carbon at the C-7a position appeared at δ 46.04. The assignments of all carbons were established through the aid of the HSQC and HMBC experiments (Table 2). Figure 3 shows the HMBC and COSY correlation of the 8, 9, 11, 12-tetrahydromecambrine (**3**).

**Table 2:** ^13^C-NMR (150 MHz), ^1^H-NMR (600 MHz) and HMBC spectral data of 8,9,11,12-tetrahydromecambrine (**3**) in CDCl_3_ (*δ* in ppm, *J* in Hz).

Position	^13^C (δ, CDCl_3_)	Type	^1^H (*J*, Hz)	HMBC (^2^*J*, ^3^*J*)
1	140.7	C_q_	-	-
1a	134.3	C_q_	-	-
1b	124.5	C_q_	-	-
2	148.2	C_q_	-	-
3	106.5	CH	6.49 s	C1a, C1, C2,C4
3a	126.9	C_q_	-	
4	27.4	CH_2_	2.92 (m) ax	C1b, C5
			2.72 (m) eq	C1b, C1a, C3
5	55.0	CH_2_	3.09 (m) ax	C1b, C4, NCH_3_, C6a
			2.45 (m) eq	
6a	65.7	CH	3.30 br s	-
7	44.5	CH_2_	2.59 (m) ax	C3a, C1a, C8, C7a, C6a
			1.75 (m) eq	C8, C12, C7a, C6a
7a	46.0	C_q_	-	-
8	34.6	CH_2_	2.14 (m) ax	C3a, C12, C7
			2.02 (m) eq	C3a, C12, C7a
9	39.0	CH_2_	2.47 (m)	C8, C11, C7a
				C8, C11, C7a
10	211.7	C=O	-	-
11	38.6	CH_2_	2.70 (m) ax	C12, C7a
			2.43 (m) eq	C12, C7a
12	36.5	CH_2_	2.50 (m) ax	C8, C11, C7
			1.93 (m) eq	C3a, C8, C11, C7a
N-CH_3_	43.5	CH_3_	2.39 s	C5, C6a
Methlenedioxy	100.6	CH_2_	5.88 *d* (*J* = 1.2)	C1, C2
(O-CH_2_-O)			5.83 *d* (*J* = 1.2)	C1, C2

Hexahydromecambrine A (**4**) was isolated as an amorphous solid, 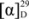 = +100 (c = 0.00003, CHCl_3_). The mass spectrum showed a molecular ion peak at *m/z* = 302.9177 [M+H]^+^, thus suggesting a molecular formula of C_18_H_23_NO_3_ (calcd. for C_18_H_24_NO_3_, 302.9167). The UV spectrum showed three peaks at 300, 245 and 265 nm, which indicate the existence of a conjugated system [37]. The IR spectrum showed a broad absorption band at 3391.78 cm^−1^, indicating the presence of a hydroxyl group. The methylenedioxyl group absorbed at 1254.36 and 929.07 cm^−1^. There was no carbonyl group present in the IR spectrum.

The ^1^H-NMR for this alkaloid showed a pair of doublet peaks at δ 5.90 ppm (*d*, *J* = 1.20 Hz) and δ 5.86 ppm (*d*, *J* = 1.20 Hz) that correspond to the methylenedioxyl group at positions C-1 and C-2. A sharp singlet peak at 6.47 ppm belongs to the H-3 on the benzene ring. The aliphatic protons of ring D resonated as multiplets between δ 1.25 to 2.45. H-6a resonated as a multiplet at δ 3.26. A strong peak at δ 2.39 appeared as a sharp peak that was assigned for a N-methyl group. H-10, which is in proximity to a hydroxyl group, resonated further upfield at δ 4.00 as a broad multiplet, suggesting the possibility of an axial configuration. Interestingly, from the COSY spectra, H-10 was determined at be pseudo-axial by the correlations H-10 (δ 4.00) with H-8_ax_ (δ 2.03) and H-11 (δ 1.75). In the case of a pseudo-equatorial hydroxyl group, H-10 will resonate at around δ 4.26–4.39 [41].

**Figure 4:**
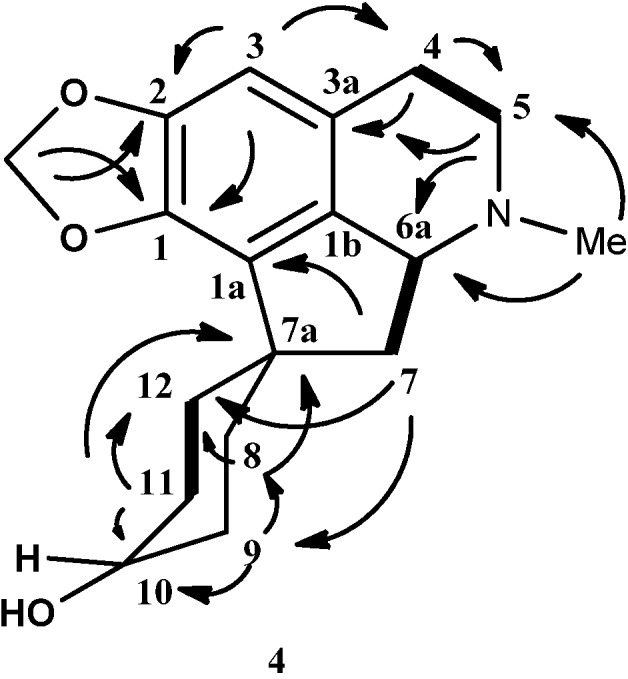
Key HMBC (→) and COSY (▬) of hexahydromecambrine A, (**4**).

The ^13^C-NMR spectrum of **4** showed the presence of eighteen carbons, which is in agreement with the molecular formula C_18_H_23_NO_3_. The DEPT spectrum showed the presence of one N-methyl, eight methylenes, three methines and six quaternary carbons. The methylenedioxyl group resonated at δ 100.5. The characteristic proaporphine quaternary spirocarbon peak C-7a appeared at δ 46.6. Apparently, C-10 resonated at δ 67.1, further strengthening the hypothesis of H-10 being axial. The complete assignments of all protons and carbons were aided by the HSQC and HMBC correlation and HMBC data were recorded in Table 3. Figure 4 showed HMBC and COSY correlations of hexahydromecambrine A. The structure of **4** resembled the structure of **3** except for the fact that the carbonyl group in **3** was reduced to a hydroxyl group in **4**.

**Table 3:** ^13^C-NMR (150 MHz), ^1^H-NMR (600 MHz) and HMBC spectral data of hexahydromecambrine A (**4**) in CDCl_3_ (δ in ppm, *J* in Hz).

Position	^13^C (δ, CDCl_3_)	Type	^1^H (*J*, Hz)	HMBC (^2^*J*, ^3^*J*)
1	148.1	C_q_	-	-
1a	129.0	C_q_	-	-
1b	131.0	C_q_	-	-
2	140.8	C_q_	-	-
3	105.9	CH	6.46 (s)	C1, C2,C4
3a	124.0	C_q_	-	-
4	27.3	CH_2_	2.93 (m) ax	C5
			2.71 (m) eq	C3a
5	54.9	CH_2_	3.11 (m) ax	C3a, C6a
			2.46 (m) eq	
6a	65.7	CH	3.26 (m)	-
7	44.3	CH_2_	2.44 (m) ax	C1a
			1.58 (m) eq	C12, C9
7a	46.6	C_q_	-	-
8	30.2	CH_2_	2.03 (m) ax	C7a, C10
			1.54 (m) eq	C12, C7a, C10
9	31.7	CH_2_	2.41 (m) ax	
			1.46 (m) eq	C8, C7a, C10
10	67.1	CH	4.00 br, m	
11	31.0	CH_2_	1.75 (m)	C12, C7a, C10
12	29.7	CH_2_	1.25 (m)	
N-CH_3_	43.2	CH_3_	2.39 (s)	C5, C6a
(OCH_2_O)	100.5	CH_2_	5.90 (*d*, *J* = 1.2)	C1, C2
			5.86 (*d*, *J* = 1.2)	C1, C2

### 2.2. Cell Culture and MTT Cytotoxicity Activity

A preliminary screening against cancer cells was performed using the MTT method [42] against MCF7 and HepG2 cell lines. The cytotoxicity of compounds **1**–**4** were assayed at various concentrations under continuous exposure for 24 h, are expressed in IC_50_ values (μg/mL), and are summarized in Table 4. Doxorubicin was used as a positive control in this study. The results showed compounds **1** and **2** exhibited cytotoxic activity against both cancer cell lines with IC_50_ values ranging from 14–60 μg/mL. Compounds **3** and **4** exhibited cytotoxic activity against the HepG2 cell line with IC_50_ values of 81 and 20 μg/mL, respectively. Compounds **3** and **4**, however, were not toxic towards the MCF7 cell line with IC_50_ values of more than 100 μg/mL.

**Table 4:** The IC_50_ values of compounds **1**–**4** and positive control on the MCF7 and HepG2 cell lines.

Compounds	IC_50_ (µg/mL) at 24 h	
	MCF7	HepG2
1	26	27
2	60	14
3	>100	81
4	>100	20
Doxorubicin	0.2	1.06

The results shown in Table 4 implied that the cytotoxic property were related to compounds with 7-oxo and methylenedioxy functions, which corresponds to the result previously described by Likhitwitayawuid *et al.* [43] that aporphine alkaloids containing a 1,2-methylenedioxy substituent appear to play a similar role in the oxoaporphine alkaloids, being required for the expression of cytotoxicity of the compounds. The aporphine alkaloid lysicamine (**1**) demonstrated moderate activity comparable to doxorubicin (positive control) against the MCF7 and HepG2 cell lines. It is important to notice that compounds **2**–**4** share the same basic skeleton with different substitution patterns. Interestingly, the proaporphine litsericinone (**2**), showed the highest cytotoxic potency against the HepG2 cell line (IC_50_ = 14 μg/mL) and MCF7 cell line (IC_50_ = 60 μg/mL) due to the presence of a methylenedioxy and a carbonyl group at the C10 position [43,44]. The enhanced cytotoxic activity of compound **4** against the HepG2 cell line implies the presence of a 10-hydroxyl in the molecule was generally results in better potency than a non-hydroxylated proaporphine. Similarly, the weak cytotoxic activity displayed by compound **3** compared to compound **2** may be due to the presence of the *N*-methyl. These findings also suggested that the aromatic ring system, 7-oxo function, and methylenedioxy ring as well as the structural planarity have powerful effects on the cytotoxic activity against different cancer cell lines [45].

### 2.3. Antibacterial Activity

The preliminary screening results of antibacterial activity against five bacterial species are summarized in Table 5. Antibacterial activities were indicated by a clear zone of growth inhibition.Compounds **1**‒**3** were evaluated for antibacterial activity using disc diffusion method. A standard antibiotic (streptomycin sulfate) was used as a positive control in the assay. Only these three compounds were tested only, as the amount of 4 available was insufficient to run the assays. The results indicated that lysicamine (**1**) exhibited a broader spectrum of activity against the microbes compared to the other two compounds—litsericinone (**2**) and 8,9,11,12-tetrahydromecambrine (**3**), which were found to be inactive.

**Table 5:** The inhibition zone diameter (in mm) of isolated compounds against selected bacteria.

Sample	Inhibition diameter (mm ± SD)				
	*Staphylococcus epidermidis*	*Staphylococcus aureus*	*Bacillus subtilis*	*Pasteurella multocida*	*Enterobacter cloacae*
	(Gram +ve)	(Gram +ve)	(Gram +ve)	(Gram −ve)	(Gram −ve)
**1**	12.00 ± 0.00	13.33 ± 0.57	15.50 ± 0.57	NI	NI
**2**	NI	NI	NI	NI	NI
**3**	NI	NI	NI	NI	NI
**4**	nt	nt	nt	nt	nt
**Streptomycin sulfate ^a^**	20.00 ± 0.00	13.66 ± 0.57	21.00 ± 0.00	21.33 ± 1.15	NI

Note: (NI)—no inhibition observed, (nt)—not tested. Doses of the samples were 1 mg/mL per disc, Streptomycin sulfate 10 µg per disc. ^a^—Positive control.

Lysicamine (**1**) displayed a strong antibacterial activity against *Staphylococcus aureus* with inhibition zones of 13.33 ± 0.57 mm, which are comparable with those of the positive control streptomycin sulfate. Moderate antibacterial activity was observed for compound **1** against *Staphylococcus epidermidis* and *Bacillus subtilis*, with inhibition zones of 12.00 ± 0.00 mm and 15.50 ± 0.57 mm, respectively. *Pasteurella multocida* and *Enterobacter cloacae* (both Gram-negative bacteria) were not susceptible to any of the isolated compounds. Generally, the results showed that compound **1** displayed strong activity against Gram-positive bacteria. The negative results obtained against the Gram-negative bacteria were not surprising, as in general, these bacteria are more resistant than Gram-positive ones [46,47,48]. These differences may be attributed to the fact that the cell wall in Gram-positive bacteria consists of a single layer, whereas the Gram-negative cell wall is a multi-layered structure and quite complex [49,50], with an outer membrane consisting of a hydrophilic surface rich in lipopolysaccharide molecules [51]. In addition, the periplasmic space contains enzymes able to degrade any exogenous molecules and also prevent the entry of inhibitors, including antibiotic molecules [47,52]. Gram-positive bacteria do not possess this type of outer membrane and cell wall structure. Therefore, antibacterial substances can easily destroy the bacterial cell wall and cytoplasmic membrane and produce a leakage of the cytoplasm and its coagulation [53]. The other two compounds **2** and **3** were not toxic towards any of the tested pathogenic bacteria since no appreciable zones of inhibition were observed. The present results regarding the antibacterial properties of the compounds from the leaves of *Phoebe grandis* (Nees) Merr. indicate that compounds from the plants of this genus could be used against the most common Gram-positive pathogen, and that their potential activity is probably due to their ability to complex with extracellular and soluble proteins and bacterial cell walls.

## 3. Experimental

### 3.1. General

Optical rotations were obtained on a JASCO P-1020 polarimeter. UV spectra were measured using Shimadzu UV-1650 PC Ultraviolet-Visible Spectrophotometer. The solvent used was methanol (CH_3_OH) while the wavelength in which the spectrum was recorded is 200–400 nm. IR spectra were recorded in CHCl_3_ on a Perkin Elmer Spectrum 2000-FTIR Spectrometer. NMR spectra were recorded in deuterated chloroform (CDCl_3_) on Bruker AVN instruments (400 and 600 MHz for ^1^H and 100 and 150 MHz for ^13^C, respectively). Chemical shifts reported in ppm on the δ scale and the coupling constants are given in Hz. Spectra signals were calibrated using TMS. Mass spectra were obtained using a Shimadzu LCMS-IT-TOF instrument. The solvent used was chloroform (CHCl_3_). Silica gel 60F, 70–230 mesh ASTM (Merck 7734); silica gel 60F, 230–400 mesh ASTM (Merck 9385); silica Gel 60GF_254_, (Merck 1.07730.1000) were used for chromatographic separations. Thin Layer Chromatography analysis was performed by using aluminium supported silica gel 60F_254_ TLC sheets (Merck 1.05554.0001) or glass supported silica gel 60F_254_ TLC plates (Merck 1.05715.0001). Mayer’s and Dragendorff’s reagents were used for alkaloid screening to identify the presence of the alkaloids and alkaloid spotting (TLC). 

### 3.2. Plant Material

Leaves of *Phoebe grandis* (Nees) Merr. (Lauraceae) were collected on 19 February 2008 from the Bukit Serting Forest Reserve, Negeri Sembilan, Malaysia and the voucher specimen (KL 5540) was deposited in the Herbarium of Department of Chemistry, University of Malaya, Kuala Lumpur, Malaysia and in the Herbarium of the Forest Research Institute, Kepong, Malaysia. The plant (KL 5540) was identified by Mr Teo Leong Eng, a botanist from the Department of Chemistry Herbarium, University of Malaya, Kuala Lumpur, Malaysia.

### 3.3. Extraction and Isolation of the Alkaloids

Dried and milled leaves of *Phoebe grandis* (Nees) Merr. (4.0 kg) were first defatted with hexane (7 L) for 3 days at room temperature, then filtered. After that they were moistened with 15% of NH_4_OH, and exhaustively extracted with CH_2_CI_2_ using a Soxhlet extractor for about 18 h. The CH_2_CI_2_ extract was reduced to 500 mL followed by acidic extraction using 5% HCI until Mayer’s test was negative. The combined extracts were then made alkaline with concentrated ammonia solution to pH 10–11 and re-extracted with CH_2_CI_2_. The CH_2_Cl_2_ fractions were washed with distilled H_2_O and dried over anhydrous sodium sulphate. The dichloromethane extract was evaporated to dryness under reduced pressure to give the crude extract.

The plant residue was extracted with methanol and the methanol was evaporated to dryness and then acidified by the addition of 5% hydrochloric acid solution and left to stand overnight. The acid solution was then filtered and made alkaline with 10% ammonia solution and reextracted with dichloromethane. The dark residue obtained after washing, drying and evaporating to dryness was added to the crude alkaloid obtained from the dichloromethane extracts to yield 13.28 g of crude alkaloids. This crude alkaloid mixture was subjected to column chromatography over silica gel using various ratios of CH_2_CI_2_ and MeOH (100:0, 98:2, 97:3, 96:4, 95:5, 94:6, 93:7, 92:8, 91:9, 90:10, 88:12, 80:20, 70:30, 60:40, 50:50) and finally with pure 100% MeOH. The collected fractions were grouped into a series of fractions, monitored with TLC. Each series were then treated separately to isolate and purify its alkaloid by extensive column chromatography followed by preparative TLC.

### 3.4. Cell Culture and MTT Cytotoxicity Assay

The MCF-7 and HepG2 cells that were used in this study were obtained from the American Type Cell Collection (ATCC). The cells were cultured using RPMI 1640 culturing media (PAA, Leverkusen, Germany). Cells were trypsinized and counted using hemocytometer and plated in a microtiter plate of 96-wells. After an overnight incubation to allow cell attachment, the medium was changed and 0.2 mL of new supplemented medium were added into each well. Cells were then treated with the different drug concentrations and incubated at 37 °C, 5% CO_2_ for 24 h. Each concentration of the samples was assayed in triplicate. The colorimetric assay were performed at an absorbance of 570 nm. Results were expressed as a percentage of control giving a certain percentage of cell viability after 24 h exposure to the test agent. The potency of cell growth inhibition for test agent was expressed as an IC_50_ value.

### 3.5. Bacterial Cultures and Disc Diffusion Assay

The *in vitro* antibacterial activity of the alkaloids was evaluated against five pathogenic microorganisms, including three Gram positive bacteria: *Staphylococcus epidermidis* (a clinically isolated strain), *Staphylococcus aureus* (S1434), *Bacillus subtilis* (B145) and two Gram negative bacteria: *Pasteurella multocida* (a clinically isolated strain) and *Enterobacter cloacae* (a clinically isolated strain). All the strains were stored in the appropriate medium before use.

Antibacterial activity of the alkaloids **1**–**3** was determined by the disc diffusion method [54] with slight modifications in terms of sample concentration, volume of sample loaded and use of paper discs. The bacteria were cultured at 37 °C for overnight in nutrient broth and potato dextrose broth, respectively. The concentrations of the cultures were adjusted turbidometrically at a wavelength of 600 nm which gave 10^5^–10^6^ colony forming units (CFU) per mL. The compounds to be tested were dissolved in dimethyl sulphoxide (DMSO) at concentration of 1 mg/mL. About 10 µL of each sample solution was loaded on Whatman No. 1 filter paper disc (Ø6 mm). The disc was placed on the surface of the agar plate (nutrient agar or potato dextrose agar) previously inoculated with bacteria. The agar plates were then inverted and incubated for 24 h at 37 °C. The antimicrobial activity was recorded by measuring the zone of inhibition (IZ) in mm around each disc, against the test organisms. The experiments were repeated in triplicate and the results were expressed as average values. The antibiotic streptomycin sulfate (10 µg/disc), was used as positive control and DMSO as negative control in the assay. Compound **4** was not evaluated due to the small amount of compound available, not enough for antibacterial screening.

## 4. Conclusions

In general, alkaloids from the leaves of *Phoebe grandis* (Nees) Mer. (Lauraceae) showed a good *in vitro* cytotoxic activity against MCF7 and HepG2 and antibacterial activity against the selected bacteria. Lysicamine (**1**) showed the most promising activity compared to other compounds in the study. This study also suggest that the presence of a methylenedioxy group, unsaturated carbonyl group, hydrogen bond donor (OH group) and dimethoxy substituent in the compounds contributes to the multiple biological activities displayed. Therefore, structure-active relationships (SAR) can further investigated to determine the potency of related compound. In a nutshell, this study can be as a basis form for selection of plant species for further investigation in drug discovery for potential new natural bioactive compounds.
